# A Treatise on Diseases of the Eye and Its Appendages

**Published:** 1836-10

**Authors:** 


### Art. II.

*A Treatise on Diseases of the Eye and its Appendages.* By Richard Middlemore, m.r.c.s., Surgeon to the Birmingham Eye Infirmary.—*London,* 1835. Two vols. 8vo. pp. 1644.

The gradual increase of institutions for the cure of ophthalmic disease, throughout the country generally, and the setting apart of particular wards in the larger metropolitan hospitals for the same purpose, have produced the most beneficial effects upon the state of our ophthalmic literature, and greatly advanced the character of British medicine and surgery. This progressive improvement, dating it from the foundation of the London Eye Infirmary, has wiped away the stigma of empiricism with which we were justly chargeable in the treatment of these maladies, prior to that period. It is true we had to boast of Cheselden, Pott, and Hey: they were, however, the stars which made the general darkness more perceptible; but it was impossible that the isolated efforts of even such men, brilliant though they were, could suffice to lay the permanent foundation of true principles, until the erection of schools of ophthalmic practice. To John Cunningham Saunders the profession owe this benefit; and it is to be regretted that Mr. Middlemore should class the founder of British ophthalmology with such practitioners as Ware, Adams, or Curtis. It is not correct to say that Saunders limited his practice, "professedly" or "secretly," to the management of ocular maladies. It is from the very circumstance of his education as a medical practitioner,—his official connexion with the anatomical school of St. Thomas's Hospital,—and his increasing avocations as a general surgeon, up to the period of his early and lamented death, that we are indebted for the successful transfer of the treatment of diseases of the eye from the exclusive oculist to the educated general practitioner of medicine or of surgery.

Having said thus much out of justice to the memory of one to whom science owes its best praise, we turn to the review of the volumes before us.

The introductory lecture of Mr. Middlemore, "somewhat rambling and discursive" as it is, and as the author very candidly admits it to be, gives an amusing sketch of the state of the science prior to the nineteenth century. At that period Germany had far outstripped us in the race; but, emulating the industry of that laborious nation, and commencing the pursuit on the true principles of physiology and pathology, we have successfully regained the ground, and are now running a fair and even course. What England was thirty years since, France now is, as regards this department of medical knowledge; and it is especially creditable to the exertions of our own country to observe the high rank her ophthalmic literature has obtained amongst our continental neighbours, while the compliment lately paid us in calling to their assistance the practical skill of one* of our most able and experienced surgeons does equal honour to their liberality and our own advancement in this department of our art.

After giving the chronology, as it were, of the science, intermixed with some fair and judicious criticism, Mr. Middlemore proceeds with general observations on the pathology of the organ of vision. He observes, "it is by due reference to the anatomical qualities of the part diseased, and the symptoms which attend, and the other events and circumstances which accompany similar morbid affections of the same texture in other parts of the body," that ophthalmic disease ought correctly to be estimated. This is the true philosophy of the science: it is by treating maladies of the eyes by constitutional remedies that the veil is torn from the charlatan, and exposes the fallacy of his "miraculous unguents and infallible collyria."

The study of this class of diseases may, moreover, be rendered of the highest utility in the investigation of the pathology of the more obscure affections of other viscera; for, small as the organ of sight may be, compared with the *organismus*, it should be recollected that it contains all the constituent tissues of the body. In this organ we are enabled to trace the origin, progress, and results of inflammation. The mucous and serous, the fibrous and cellular membranes are displayed before us. We can trace the commencement of an inflammatory attack, the impairment or destruction of the tissues involved, the action of the remedies, and the result of the disease or of our opposing remedies. This cannot be without its applicability in the wide range of hidden disease. The student cannot behold the treatment of ophthalmic maladies without being

* Mr. Lawrence.

perpetually reminded that similar tissues elsewhere are subject to like attacks, and curable by the same general measures.

Mr. Middlemore's volumes are strictly devoted to the pathology and therapeutics of eye diseases, mixed up, however, with some anatomical details, which we shall deem it our duty to notice. Upon this important subject we have had, within the last four or five years, the works of Mackenzie, Lawrence, Guthrie, and Walker. These productions are all of them highly creditable to the authors individually, and reflect much lustre on the profession to which they belong: the two former ones especially evince great powers of observation, patient and laborious and learned research, originality of thought, and a clear and lucid style. The success which attended these publications might have deterred, for a time at least, any new competitor for ophthalmic laurels from appearing in the field. We have scarcely, however, had breathing time before a rival has started up in the shape of two huge volumes, a great part of which is made up of quotations, or rather lengthened extracts, from a host of writers, whose names and ideas are used with so much freedom as often to leave us at a loss for the author's own opinions.

We are, however, unwilling to criticise this work by a studied comparison of it with others; neither is it just so to do. It must stand or fall by its own merits; and it is only when we see Mr. Middlemore attempting to depreciate others, that we feel called upon to add our voice in condemnation of such a practice. We are therefore exceedingly sorry when we see the flippancy with which Mr. M. attacks the opinions of those with whom he may happen to disagree; as, for instance, in his remarks on Mr. Guthrie's observations respecting the insensibility of the eye, and the surgical liberties that may be taken with it.

Another cause of complaint we have against Mr. Middlemore is his too great haste to appropriate to himself certain methods of treatment and explanatory views of obscure pathology. At page 70, vol. i., he appears very anxious to take the merit of having introduced the sulphate of quinine as a remedy in scrofulous affections of the eye. He does not give us the date of his discovery; but, if we remember rightly, in November, 1828, Dr. Mackenzie noticed the subject in the Glasgow Medical Journal, where he states the beneficial effects of quina in phlyctenular ophthalmia, strumous corneitis, and ophthalmia tarsi. We should hardly have noticed this fact, had not Mr. Middlemore evinced considerable soreness at some criticisms of the editor of the Medico-Chirurgical Review. Mr. M. should remember that, however elegant a form of tonic the sulphate of quina may be, and by whomsoever introduced, the principles upon which the practice is founded were known and pursued long before his time. Drs. Fordyce and Fothergill, in the first volume of the "Medical Observations and Enquiries," recommended the use of bark in cases of strumous inflammation of the eyes, as long back as the year 1755.

The explanation which Mr. Middlemore gives of the sympathy existing between the lachrymal gland and the conjunctiva is almost identical with that given by Mackenzie in the first edition of his "Practical Treatise." (Compare p. 259-260 of Middlemore with Mackenzie's first edition, p. 394-5.)

We now turn, with much greater pleasure, to those portions of these volumes wherein we have little fault to find, and much to praise. The pathological portion of the work opens with the subject of "Conjunctivitis," and, as that malady is familiar to every one, an extract will enable our readers to form a more correct estimate of the author's powers of description, than if we were to select some of the more obscure, and because obscure perhaps more interesting, forms of disease.

We have first an outline of acute conjunctivitis, light and sketchy; the more prominent symptoms are then filled in,—the redness, pain, lachrymation, the extension of the disease to other tissues, and the results, are all amply dilated on. Then follows the diagnosis, which we give entire.

" *Diagnosis.* The only disease of any other parts of the eye with which acute inflammation of the conjunctiva is liable to be confounded is sclerotitis. You would distinguish the disease under consideration from inflammation of the sclerotica by the colour of its vessels, which, you know, are bright scarlet, whilst those of the sclerotica are of a purple appearance, from their situation: they are placed upon and within the conjunctiva, and may be moved with it upon the eyeball; those of the sclerotica are deeper seated, the conjunctiva may be moved upon them, whilst they only follow the movements of the eyeball :—from their arrangement; they are first perceived at the periphery of the eyeball, and are most abundant there; those of the sclerotica are first observed near the margin of the cornea; and, finally, the vessels of the inflamed conjunctiva are less direct, more tortuous, than are those inflamed sclerotica.

" In saying that the vessels of the inflamed conjunctiva are first perceived at the circumference of the eyeball, and subsequently extend to and ramify around the margin of the cornea, I ought to mention that this is usually noticed, but sometimes the progress of the disease is too rapid; the symptoms of the affection are established too suddenly to furnish us with an opportunity of doing more than just notice that the largest vessels and the greatest number of them are placed at the periphery of the globe, at an early period of the malady. When the disease is fully developed in its most acute form, the conjunctiva presents one uniformly and equally red surface; or, rather, it presents a vividly bright vermilion appearance. You will also distinguish inflammation of the conjunctiva from sclerotitis by other symptoms; for, in the latter disease, the dryness of the eyeball, the uneasiness produced by moving the lids upon it, and the pricking or scratching sensation, as though sand were beneath the lids, is either not noticed or scarcely at all perceived ; whilst, in conjunctivitis, these symptoms are present to a distressing extent; and, on the contrary, the pain, the sense of tension and intolerance of light, are peculiarly severe and annoying in inflammation of the sclerotica, whilst they scarcely provoke the slightest uneasiness in conjunctivitis.

"I have already told you that inflammation of the conjunctiva may extend to the sclerotica; and it is right that you should be informed that, although I am describing the diseases of each texture of the eye separately, yet you will not always find them so limited in the course of your practice; but, if I were not to adopt this plan, you would be quite unable to comprehend and distinguish the numerous maladies to which the organ of vision is liable. If I first describe the symptoms of each form of disease of the separate textures of the eye, and subsequently point out those which are usually associated with its more common combinations of morbid affection, you will be prepared, I trust, when you witness such combinations of disease, to attach to each malady the symptoms which belong to it, to select the symptoms connected with separate maladies, and attach to each of them their proper share in producing the aggregate disease, so as to distinguish the predominant affection, and educe the comparative date and severity of each. I repeat, then, that when the conjunctiva is acutely inflamed, the sclerotica is very liable to participate in the disease, chiefly from its contiguity and the vascular connexions subsisting between these parts; and, in such case, you will be able to distinguish the various circumstances connected with the colour, situation, arrangement, and so on, of the two sets of vessels, with all the advantages of contrast. In such an instance it will be scarcely possible to confound the moveable, prominent, scarlet vessels of the conjunctiva with the fixed, purple, covered vessels of the sclerotica.

Having said so much respecting the means of distinguishing the inflammation of the conjunctiva from that of neighbouring parts, it will only be necessary, in order to complete the subject of diagnosis, that I should just mention that the other varieties of conjunctivitis are distinguished from that under consideration, by the quality or quantity of the discharge proceeding from the inflamed surface or by other sufficiently distinctive symptoms, so that they are only liable to be confounded by an extremely ignorant or a culpably inattentive person." (Vol. i. pp. 45-48.)

The symptoms in the above-quoted extract are minutely detailed and judiciously discriminated, and the allusion the author makes to the plan he has chosen to follow affords the best apology for the size and diffuseness of the work.

Six pages are devoted to the causes of acute ophthalmia, and then follows the treatment. All this is ably done, and Mr. Middlemore here takes the opportunity of speaking more at large upon the subject of the measures necessary to subdue ocular inflammation generally. Thus, the much-abused or misused bloodlettings pass under review. Several long quotations from various authors are introduced; Vetch, Lawrence, Thomson, Langenbeck, Mackenzie, and others are adverted to; yet little practical is deduced from all this. We are informed, indeed, what others recommend, we see the discrepancies of opinion, but are left to collect the author's opinions from the following reference to his own practice.

"It must be understood, that no directions you can receive from books or lectures will enable you to decide upon the quantity of blood it may be necessary to withdraw in every case of acute inflammation of the textures of the eye; there must be a demand, a very great demand, on your own judgment.

"I cannot tell you that the loss of so many ounces of blood will be necessary to subdue a certain degree of inflammation of the conjunctiva, and that the abstraction of so many more or less number of ounces will be required to remove inflammation of the sclerotica, and so on, with regard to the inflammation of the other textures of the eye. However, the following directions comprehend the rules which regulate my own practice: you must be guided by the effects of bleeding upon the constitution, as well as by its influence upon the eye; you must bleed repeatedly in a very short space of time, if symptoms are severe; for, as I may again remark, unless you abridge the duration of acute inflammation, unless you check its progress with promptitude, interstitial deposition may take place, and you may experience the disappointment, and your patient may sustain the injury, of recovering the form of the eye perhaps, but with the loss of the transparency of its pellucid textures, after having cheerfully submitted to that treatment, which, if carried a slight degree further, would have perfectly preserved both its figure and its transparency." (Vol. i., pp. 71-2.)

From what we have here quoted it is evident that Mr. Middlemore does not fear the use of the lancet, although he does not run into those lamentable extremes by which the patient has often been drained almost to the last ounce of his blood, and left obnoxious to all the forms of cachectic disease. We are convinced that "venesection ad deliquium" is, as a general rule, deserving the highest degree of censure. In no case of ophthalmic disease that we are acquainted with, not even in the purulent ophthalmia of adults, can a bleeding to fifty, sixty, or more ounces be productive of anything but mischief. The inflammation is not subdued by such treatment, but only converted into a passive process, often equally formidable and more unmanageable than the original attack. The chemosis, in such cases, far from diminishing, appears to bury and constrict the cornea more closely as the powers of the patient fail under the lancet; and that transparent tissue sloughs as much from the vitality of its structure being reduced by loss of blood as by strangulation of its vessels. We observe, however, by the description of the treatment of purulent ophthalmia in Mr. Middlemore's work, that he adopts a cautious plan of procedure, although his rules of practice are not altogether clearly explained.

The subject of Iritis is, upon the whole, well treated of by the author. From amongst the general symptoms described at page 618, we would strike out Nos. 2, 3, and 4; because dimness of vision, intolerance of light, and increased lachrymation are equally symptoms of other inflammations of the eye. Nos. 1, 5, and 6, will then stand as general characteristics of idiopathic iritis. The detail of symptoms is very good; but, when upon the subject of the contraction of the pupil, Mr. Middlemore starts into a theoretical discussion, which he must pardon us if we state rather obscures than throws light upon the matter. Until the question of how the iris is moved,—if by muscles, to what laws they are subjected, &c.,—be finally settled, it is obvious that any attempted explanation of this abstruse point would be nugatory. We cannot indeed see that "useful objects are attained by this contraction of the pupil" (p. 625,) in iritis: on the contrary, if the pupil remain in this condition for but a very short space of time, we have to fear all the disastrous consequences of inflammation of a serous tissue,—effusion of lymph, permanent irregularities or occlusion of the pupillary aperture. On the other hand, it is of the highest importance not only to dilate the pupil with belladonna, in order to obviate these results, but also to effect that dilatation in the highest possible degree, since the distance of the margin of the iris from the anterior surface of the crystalline capsule is greater in proportion as it is opposite to that point where the lens is bevilled off to its sharp circumference. Thus, when the lens is kept in a medium state between contraction and dilatation, adhesions may still take place; and, although a permanent aperture be secured for the transmission of the rays of light, still, the freedom of motion being destroyed, the vision is proportionally impaired.

In speaking of the cure of iritis, the author gives a clear and full explanation of the means to be most certainly relied on; as bloodletting, mercury, and the local application of belladonna. With these remedies in our hands, there are perhaps few cases of iritis that may not be said to be perfectly manageable, provided always the case comes under observation before irreparable mischief be done by the long continuance of adhesive inflammation. We are inclined to think with Dr. Farre, whom the author has himself blamed, that with mercury alone the disease may be effectually cured. Dr. Farre stated, in the introduction to Mr. Saunders's posthumous work, at the very place which has called forth Mr. Middlemore's expression of "injudicious," as applied to Dr. F.'s opinion that his views of the action of mercury were "the result of a fair trial." We have seen, in the practice of the surgeons of the London Ophthalmic Infirmary, repeated instances where iritis of the severest kind was treated, and with success, solely by the use of calomel and opium, accompanied by the application of the extract of belladonna. We would further undertake to say, of all the cases occurring there, either of acute or syphilitic iritis, that greatly more than three-fourths of them are so treated. The extensive opportunities enjoyed by the medical officers of that institution render the result of their practice of more than ordinary weight.

On the subject of Glaucoma, Mr. Middlemore quotes Mackenzie's observations on the pathology of this disease, evidently from the first edition. We would wish to refer him to the second edition of that valuable work, wherein he will find the subject somewhat differently treated, especially as regards the morbid condition of the crystalline lens: indeed, what Mr. Mackenzie expressly says he considers a chief cause of the glaucomatous appearance, namely, the amber or reddish brown colour of the posterior lamellæ of the lens, is wholly omitted. We do not think, either, that Mr. Middlemore is justified in affirming that "Mackenzie differs from almost every preceding writer in stating that the well-known appearances of glaucoma (those peculiar appearances as respects colour and opacity which are presented on a close examination of the eye,) are independent of any opacity of the vitreous humour and change in its colour." Transparency may exist with change of colour; and Mr. Mackenzie expressly says, this fluid was perfectly pellucid, colourless, or *slightly yellow.*

In the description given of Senile Glaucoma, Mr. Middlemore's explanation is almost identical with that given by Mr. Dalrymple, in "The Anatomy of the Human Eye," with the exception that Mr. M. does not allude to the diminished secretion of the pigment, which, to a great extent, accounts for the reflecting instead of absorbing surface at the bottom of the eye.

"The vitreous body is more fluid and less transparent in the aged than in the adult. Owing to the diminished secretion of the pigment and its lighter colour, combined with the opaline tint of the lens, the pupil, instead of presenting the intense blackness of the earlier periods of life, is often, in age, of a greenish hue. This has frequently been confounded with the disease called "Glaucoma," while the thinness and loss of energy in the retina itself aids the deception by furnishing, on the consequent failure of vision, one of the symptoms of that distressing malady. I am inclined, however, rather to look upon such appearances, in very old people, as indicative of a natural change in the organ, than as a true morbid affection, unless accompanied with other and unequivocal symptoms." (*Anatomy of Human Eye*, 1834.)

After stating his belief that the changes which produce what is called senile glaucoma are the natural alterations of the ocular tissues, (which ought to take it out of the catalogue of diseases,) Mr. Middlemore builds up a theory upon very slight foundations, and which we think he will have some trouble to substantiate, viz. "that the secretion and absorption of the vitreous humour are equally prevented," and that *for a useful purpose:* or, in his own words, to prevent "the flaccidity and flattening of the globe," and consequent failure of the focal powers.

As an operating surgeon, Mr. Middlemore must, however, have remarked there is no want of secretion of the aqueous fluid in old persons, at least, after the operation of extraction, as is attested by numerous cases where that mode of removing cataract has been performed on patients between the age of eighty and ninety years, or even upwards. It is not because we deny secretion to be feeble in the aged, that we do not agree with the view Mr. M. takes of these changes, but on account of the reason given for it. If it were to prevent certain inconvenient results that secretion and absorption are stayed in the eyes of old people, senile glaucoma would be a constant occurrence and a beneficial change, and not an affliction.

We have but little space remaining to extract from this voluminous work, (so large indeed as to defy systematic criticism,) yet we wish to allude briefly to the subject of dislocation of the lens, for the purpose of introducing a very interesting case which concludes the section. The causes, symptoms, and appearances of this affection or accident are well described. Reference is made to three cases of displaced crystalline, where that body retained its transparency; the question, indeed, whether the lens should or should not become opaque in its abnormal situation, according to our present imperfect knowledge of its organization and nourishment, is very wisely not discussed. It is well known that, in the vast majority of instances, the lens, when dislocated, acting as a foreign body, produces great excitement in the eye, and sets up a degree of inflammation that, uncontrolled, inevitably terminates in total blindness. Nor is this all: the agonizing pains, both in the eye and surrounding the orbit, call imperatively upon the surgeon for some instant relief. This, we should naturally conceive, might be effected by removing the cause, by extracting the lens; but Mr. Middlemore justly observes, that great care and discrimination are necessary to determine how far the symptoms are dependant upon the presence of the crystalline or upon the inflammation "excited by the original injury, of which injury the dislocation of the lens is merely a part." For our own opinion, we believe it is advisable to remove the lens, if possible, before inflammatory action be set up; and it may, moreover, become a question whether, when such action is excited by the injury itself, the removal of the foreign body would not tend in a great measure to allay that disturbance which its presence only aggravates. If the operation be determined upon, we should not wish to follow Mr. Middlemore's directions of carrying the knife *behind the lens*, inasmuch as the iris is more likely to be wounded by the edge of the instrument, which is then concealed from our view by the interposed body; and because of the difficulty of insinuating the knife between it and the iris without implicating the latter structure. The section, we hold, should always be of the upper half of the cornea; since the lens will, in that case, gravitate to the bottom of the anterior chamber, leaving a freer space for the passage of the knife.

The case which concludes the chapter is of considerable interest, one in which the lens remained some months in its dislocated position without its transparency becoming impaired. We have at this moment under our observation a case in which the lens remained for upwards of three months in a similar transparent condition, and ultimately only begun to become opaque from accident. This was a case in which, as in Mr. Middlemore's patient, the lens appeared to have been spontaneously displaced; at least, rather the result of internal action than external violence.

"*Case.*—A man, who had some years previously lost the sight of his right eye, discovered a considerable confusion of vision in the left, which came on without any assignable cause, and soon increased so much as to render him unfit to follow his employment. At this period he applied to Mr. Smith, of Southam, for relief. On an examination of this recently affected organ, it was found that the lens had become dislocated, and floated about sometimes in the posterior and at others in the anterior chamber. The patient remarked on this point, that, when he stooped forward, the lens projected; that is, it fell into the anterior chamber, and pressed back the iris, producing slight uneasiness: it was, in fact, quite under the influence of gravity; it was also seen that the crystalline humor preserved its figure, magnitude, and transparency, and it was said by one gentleman who examined the case, to resemble a drop of clear oil floating on water. In this condition he came to the infirmary, three months after he had first discovered the malady; he had then no useful degree of vision, although his sight varied much, and chiefly in accordance with the position of the lens. As there was no inflammation present, as the displaced lens excited no irritation, and as the eye was considered to be affected with incipient amaurosis, my colleagues thought, and I was of the same opinion, that it would be quite wrong and unnecessary to extract the crystalline body." (Vol. ii., p. 49.)

Mr. Middlemore has not pursued a course with the anatomy of the eye similar to that adopted by Mackenzie and Lawrence, inasmuch as there is no introductory chapter expressly devoted to the subject. It is true, we do not think that a sufficient degree of knowledge can be acquired by the student from the space usually given to the anatomical details in works essentially pathological; but, as far as the subject is treated of by Mr. Middlemore in the proems attached to each section of disease, we feel that he falls very short of the accuracy and minuteness of the above-mentioned authors. We fear, indeed, that he has studied the writings of the illustrious anatomists whose discoveries he mentions at page 6, to very little purpose. Had he been more acquainted with their works, he would have been aware that, with but one exception, none of the parts in question (Liquor Morgagni, Canalis Petitiana, Foramen Soemmeringii, &c.) received the appellations by which they are now known, from the discoverers themselves. The modesty which accompanies true merit was (Ruysch excepted,) a distinguishing characteristic of these men. It was the consenting voice of their contemporaries or successors that rendered their names as imperishable as the science they adorned.

Mr. M.'s ideas as regards the anatomy of the internal membranes of the eye seem to us inaccurate and unscientific. "The choroid,

(he says,) is placed between the medullary or pulpy layer of the retina and the sclerotica," (page 770;) and again, when speaking of the retina at page 789, "It (the retina) consists of two parts, the medullary, *which is opposed to the choroid*, and the membranous," &c. From this it is clear that Mr. M. is a sceptic as relates to the tunica Jacobi, especially as he goes on to observe, in a very depreciating tone, "that, in addition to this vascular layer and its medullary layer, Dr. Jacob has fancied he has discovered a third layer or membrane situated between the medullary layer and choroid." But we must remind Mr. Middlemore, that this fancy of Dr. Jacob is participated in by all the best anatomists of Europe, and that it was by the unanimous voice of scientific men that the honour was accorded of affixing Dr. Jacob's name to this tissue.

Mondini's membrane of the pigment has met no better fate at Mr. Middlemore's hands; for we find him expressing a belief that, after having washed off the pigment, (and with it, of course, Mondini's membrane,) the choroid "more nearly resembles the serous than any other class of tissues." We should be glad to know wherein the similitude consists. Where is there any reflexion of that membrane? Where the closed sac of serous tissues? What serous membrane in the whole body is found secreting, as he asserts the choroid does, a dense, opaque, and pasty pigment?

Without going further into this subject, we may say, from the specimens before us, that we are sorry Mr. M. introduced any anatomical details whatever, as they neither add to the value of his book, nor to his reputation as an accurate or minute observer.

In conclusion, we beg to express our regret at having had occasion to dispraise so many portions of this work. Notwithstanding the sanction it has received from the College of Surgeons, who awarded the Jacksonian prize for the year 1831 to what, we conceive, was the original draft of this treatise, we are bound in all fairness to state that we are convinced it would be advantageous to condense the two volumes into one. This might be easily done by expunging many of the long extracts from other writers, and by avoiding a prolixity that is often very wearisome to the reader. There is a quaintness of expression, also, that frequently grates harshly on the ear; although we would not quarrel with the style, were the matter conveyed original and to the purpose.

